# Dr. Edward Jenner on the Influence of Artificial Eruptions in Various Diseases

**Published:** 1822-06-01

**Authors:** 


*A Letter to Charles Henry Parry, M. D. F.R.S. &c. &c. on the Influence of Artificial Eruptions, in certain Diseases incidental to the Human Body, with an Inquiry respecting the probable Advantages to be derived from further Experiments.* By Edward Jenner, Esq. M.D. LL.D. F.R.S. M.N.I.F. &c. &c. &c. and Physician Extraordinary to the King. Quarto, pp. 67. London, 1822.

Counter-irritation, as a remedial measure, is one of the oldest and best established principles in medicine. We every day witness it employed by Nature—we every day act upon it, either designedly or undesignedly. Thus we see spontaneous eruptions on the skin relieve internal disorders—and irritations in the bowels, inducing diarrhœa, carry off affections of parts situated at a great distance from the alimentary canal. These phenomena must have led careful observers, in the earliest ages, to imitate nature by art. Accordingly we inflame the skin by blisters or other irritants when disordered action or pain is going forward in a deep-seated part—and we irritate, by emetics and purgatives, the internal surface of the body (the stomach and bowels) on the same principle, when either the skin or any other part than the alimentary tube is in a state of morbid irritability.

The ancient observation—"ubi stimulus ibi affluxus"—has been amply illustrated and explained, in our own times, by Dr. Parry, under the terms natural and artificial "*determinations*" to particular parts of the system, as will be seen in the first article of this number. In short, the principle of "derivation," or "counter-irritation," is that which is the most widely employed by all classes of practitioners, of any principle in pathology or therapeutics.

The illustrious discoverer of vaccine security has long directed his attention to the effects of *pustular eruptions*, artificially excited, in many diseases incidental to the human body, and has now laid before the public an ample collection of facts and histories for the illustration of those effects. We shall present our readers with short analyses of many of these cases, as the quarto form of the publication will limit its circulation through the minuter ramifications of the profession.

*Case* I. A man, 60 years of age, originally a seaman, had been occasionally affected with sick head-aches, but otherwise in good health. A disappointed scheme threw him into a state of hypochondriasis, which ended in decided insanity, in about three months from the commencement of the hypochondriacal affection. He went through the ordinary routine of treatment, of bleeding, purging, and antimonials; but the disease continued, and the bowels were so obstinate, that it was almost impossible to get any passage through them. A drachm of tartarized antimony, incorporated in an ounce of simple cerate, was now rubbed on the insides of the arms, from the elbows to the wrists, night and morning. Papulæ of some magnitude were produced, and the transition from derangement to health was inconceivably rapid. The cure was permanent. The next case related is of a similar description, and with the same result.

*Case* III. A youth, ætat 17, was found by our author apparently in the last stage of pulmonary consumption, the symptoms of which are too well known. In addition to the usual phenomena, however, "there was a perceptible enlargement about the centre of the left side of the thorax." To engage his mind, and without any hopes of ultimate recovery, the antimonial ointment was rubbed on the protuberant part until pustules were produced, which was effected in a few days. In a week there was some amendment in regard to the cough and other distressing symptoms. The application was continued with the view of keeping the pustules in full activity. At the expiration of a fortnight the general bad symptoms had considerably abated, and the patient's looks were much improved. From this time the convalescence was so rapid that, in six weeks more, no vestige of disease remained, and the youth began to renew his ordinary avocation, that of a stone-cutter. Dr. Jenner very properly wishes that it may not be inferred from this case that he supposes genuine confirmed phthisis is to be cured by artificial eruptions.

The fourth case was a woman, 54 years of age, subject to spasmodic asthma. She used the ointment on the nape of the neck, after which the complaint was more slight, and the intervals longer.

*Case* V. A seaman, 47 years of age, was suddenly seized with inflammation of the right eye, but not so violent as to prevent him pursuing his avocations for nearly three weeks, when a chill came on daily between three and four o'clock.

"About half an hour after each attack, a pain seized the right side of the head, principally about the orbit of the eye, extending in the course of the temporal muscle. It continued to return every afternoon at a certain hour, and at length became so violent as to deprive him of sight and intellect. In one of the paroxysms he grew enraged with his wife, because he supposed that she had not lit him a candle, although one was burning before him. These periodical attacks became marked in the end with raving madness. In a paroxysm, with extreme severity of pain, he was at the point of destroying one of his children. He was bled from the arm, leeched, and took purgatives up to drastics, but they took no effect upon the malady. Seeing the impression which tartar emetic had made on affections connected with a disordered state of the brain and nerves, I did not hesitate to direct the application of the ointment, and it was applied to the left arm. Pimples followed in twenty-four hours, and as soon as they became acuminated, and *contained a little limpid fluid*, the patient found ease; the pain continued to abate, and at the end of *three days it was quite gone.* He continues well. This man, like many others who ply as mariners on our river, (the Severn,) was a hard drinker." P. 9.

*Case* VI. The sixth case was a boy, 12 years of age, who had been ill six months, with what is called chronic hepatitis. His liver felt hard, enlarged, and very sensible to the touch. The cachectic appearance and general emaciation were such as indicated the probability of a fatal termination. At this period the ointment was applied, with the usual cutaneous effect, "after which he recovered with astonishing rapidity, and no vestige of the induration or enlargement within the abdomen remains." Mild aperients were used during his indisposition.

*Cases* VII. VIII. IX. The seventh and eighth cases are equivocal, or unsatisfactory. The ninth case was that of a young woman with mania, the patient of Mr. Fewster of Thornbury. Her ravings were violent. Leeches were applied about the head, and the ointment upon the leech-bites. "As soon as vesicles appeared, she was well." Having neglected to continue the ointment, she experienced a relapse, and became decidedly maniacal. The ointment was reapplied, and she soon recovered.

*Case* X. (A patient of Mr. Fry of Dursley,) Mrs. B. ætat 23, was seized with puerperal mania the second day after parturition, and became totally unmanageable, refusing food and medicines. The antimonial ointment was rubbed along the inner surface of the forearm, from the joint to the wrist; but a fortnight elapsed before a vesiculated eruption was brought out. "As soon as the eruptions appeared, amelioration of her symptoms was evident." She was kept under the influence of the medicine nearly three weeks, during which time she progressively improved. During the external applications she took occasional purgatives, and a solution of tartarized antimony internally in nauseating doses. Considering that this was *puerperal* mania, and that the other means were pretty efficient, we can hardly agree with our worthy author, that "her restoration may be chiefly imputed to the ointment."

*Case* XI. The eleventh case was insanity unconnected with pregnancy. The disease was removed by the ointment; but the patient relapsed, and when the work went to press, her friends were endeavouring to get her into Saint Luke's.

*Case* XVI. We shall pass on to the 16th case, the intervening ones not being particularly interesting.

Mr. F. S. ætat. 37, a hard drinker, caught cold in an open carriage, which was succeeded by hæmoptoe. His respiraration, at the time of report, was very quick and difficult, with cough, and expectoration of viscid phlegm. Has been bled repeatedly during his illness, now of thirteen weeks duration, and with some relief. Last of all he used the ointment, which excited a very irritative crop of pustules on the chest. When they were fully formed, and had discharged, the patient found great relief of respiration, but not so decidedly of the cough. In another month the patient was farther improved, but the account breaks off, when he was very far from well.

*Case* XVII. This was a man, 40 years of age, apparently phthisical for 25 years past, and subject to attacks of hæmoptoe.

"After being severely indisposed with affections of the chest, *viz.* cough, and impeded respiration; he was seized, last summer, with a dangerous recurrence of the hæmorrhage. It was concluded that he had pulmonary consumption in the last stage, and the case afforded every indication of terminating fatally, but the hæmoptoe, which became more and more violent, was at last arrested by super-acetate of lead, and the tart. emet. ointment was applied to the chest. As soon as the eruptions vesiculated, he got better; when they died away, he began again to feel uncomfortable about the chest, complaining that he felt "plugged up in breathing." He finds immediate ease by a renewal of the eruptions, and has, in consequence, continued under their influence for nearly twelve months past. His skin is so irritable, that pimples almost immediately follow the application, though in some individuals three days will elapse before they will be excited. The ointment gives him most relief when applied to the opposite side of the chest to that most affected." 23.

*Case* XVIII. We marvel much that our able and intelligent author did not place the following cure to its real cause —the removal of obstruction from the hepatic duct, rather than the application of antimonial ointment. We shall give the case entire, that our readers may judge for themselves.

"John Gay, 39 years of age,—Was taken ill about four months ago, with feelings of languor and nervous debility, accompanied with dyspepsia, bilious and acid eructations: he had also pain in the right hypochondrium, dry sore throat, and general feverishness. With these symptoms he had pyrosis. His complaint continued to grow worse, especially a dull pain which had been going forwards in the region of the liver, till he was seized with symptoms of complete obstruction of the common duct of the gall-bladder. His skin became tinted with a deep blackish yellow colour. The food and medicines which he took, for some time after the symptoms of of obstruction, regurgitated in an unaltered state, from the stomach.

He found great difficulty in procuring medicines, from different medical men, that would act on his bowels. In this emergency, scanty evacuations of slime were procured by the administration of clysters; but he passed no solid stercoraceous stools; pills of soap and rhubarb, combined with an aromatic, and also the ointment, were now prescribed for him. Pimples appeared within twenty-four hours. These suppurated, and discharged pus with unusual freedom, and disposition to continue to discharge. About the time at which the pustules appeared, a sudden burst of evacuations took place, consisting first of bilious coloured fluid, next of slime of a green hue, and an abundance of shreds of a skinny appearance. In his first stools, at this time, my patient observed a mass, which he conceived to be food and medicines which he had taken previously, and had remained unaltered in the alimentary canal. The pain in his right shoulder and right hypochondrium abated rapidly, and his stools came away more solid, but still enveloped in slime. He is now convalescent, but tender under the margins of the right ribs, and possessing a mitigated degree of unhealthy action about the liver. As his health *improved*, the eruptions evinced a *disposition to dry up*, but they have been continued. He had, four years ago, a constitutional sore, which has occasionally healed and re-appeared: its final suppression may have had some connexion with his present complaint. His recovery went on and was perfect in far less time than I could reasonably expect, considering the extreme state of debility to which he was brought by his long and severe sufferings. Within six weeks he resumed his laborious occupation, which was that of a sawyer." 24.

We were lately in attendance on a remarkable case of jaundice from simple obstruction of the ducts, some particulars of which may not be uninteresting. A young medical gentleman in the city had been attending a bad labour—at first protracted, then with retention of the placenta, and afterward, we believe, uterine hæmorrhage. The fatigue or anxiety—or both combined, produced jaundice in about 48 hours after the accouchement. Dr. B. became quite yellow, but continued to go about his professional avocations, complaining of lassitude and all the usual symptoms of jaundice, with very yellow urine and white stools. The jaundice continuing, and the tint deepening for ten or twelve days, with loss of strength and appetite, the patient and friends got alarmed, and we were requested to see the gentleman, with Dr. Farre, and occasionally Dr. Babington. There was at this time no pain in the side or at the pit of the stomach; but the skin was intensely yellow, the stomach irritable, the stools white, the mind desponding, and the physical powers all depressed. The usual remedies were tried, as the warm-bath—mercurials, aperients, local bleeding from the region of the liver, &c. &c. but all without the slightest advantage. In short, the complaint went on nearly seven weeks, the patient getting weaker and more emaciated every day, with great gastric irritability. Some of the attendant physicians considered the case as very dangerous, the obstruction being looked upon as of some fixed or organic nature, which would terminate by effusion in some of the cavities. About the middle of the seventh week, however, when great despondency prevailed on all sides, Dr. B. complained of pain, for the first time, at the pit of the stomach, which gradually but rapidly increased, till it ended in the tortures of gall-stones. In about 24 or 30 hours from the commencement of the pain, vomiting, and spasms, a burst of bilious fæces came away, with cessation of pain, and, of course, an end of the jaundice. This took place in the night, and the servant threw away the first two or three motions, so that the biliary concretions were not detected, but no doubt could exist as to their having been past by stool.

The above case is highly important in many respects. In the first place it shows how quickly mental anxiety will produce jaundice by obstructing the biliary ducts, probably from spasms of their mouths, whereby the bile stagnates in the passages, and becomes inspissated there. In the second place it teaches that complete obstruction to the natural course of the bile—in other words, simple jaundice, may continue many weeks without being fatal. We have seen but one other case where jaundice from gall-stones continued so long, and ultimately did well. In the case here alluded to, which was also that of a medical man, the obstruction remained thirteen weeks, and then terminated by painful expulsion of gall-stones. In general, however, simple jaundice lasting longer than two or three weeks, becomes a dangerous disease, and requiring a very guarded prognosis. In old people it is still more dangerous than in young. In organic diseases of the liver, as where a tubercle presses on and obstructs the ductus communis, jaundice will last many months without proving fatal. We are at this time (April 1822) attending with Mr. Brien, an intelligent surgeon of Spencer Street, a man who has been intensely jaundiced for more than ten months. He has organic disease of the liver, probably tubercles, and is greatly emaciated, but not yet dropsical,

To return to the case of our young medical friend. It appears either that the obstructing cause lay dormant in the duct for so many weeks, like a fœtus in utero, until its size or other circumstances induced expulsive efforts in the duct; or else the cause was gradually and slowly advancing to the extremity of the tube, and did not cause pain till it came to its mouth, which is known to be much more sensible than any other portion of the canal. In either case, we apprehend that we have very little power over the mechanical obstruction. In the case under consideration, every mean was tried in vain—including impregnation of the system with mercury—emetics—electricity—warm baths—soap and alkalies —purgatives—frictions of mercurial ointment, &c. &c. &c. After we had ceased to administer remedies, the expulsive nisus in the duct came on spontaneously, and the concretion was dislodged. But we may here mention that we have seen some instances of jaundice from mental affections, where the cause was very quickly removed, (at a very early period of the complaint,) by administering five or six grains of calomel and two grains of opium over-night, and next morning giving the patient a brisk emetic. We pursued this plan on the supposition that spasmodic constriction of the biliary duct is the first link in the production of biliary concretions, and consequently jaundice; and that the opium and calomel tended to relax this constriction and increase the *vis a tergo*, or secretion of bile. The mechanical action of vomiting was employed for very obvious reasons. We have to apologise for this digression; but hope it is not entirely devoid of interest.

We have now presented our readers with an analysis of those cases which Dr. Jenner has laid before the profession. The trials, as he acknowledges, which have been made of exciting vesiculo-pustular eruptions, are as yet limited, "but he trusts the general favourable results of the experiments made, will apologize for his hazarding a few physiological hints for the consideration of those who may think a wider basis to work upon desirable." These hints are chiefly speculative, and founded on his "favourite pedestal analogy."

It appears that about 40 years ago two papers were published on the subject of the external application of tartrite of antimony—the one by Mr. Gaitskill, an able and experienced surgeon at Rotherhithe;—the other by Dr. Bradley. The latter gentleman relates some interesting observations respecting the use of the remedy in rheumatic affections. In every instance it appeared to Dr. Bradley to be a remedy of great efficacy; but the disagreeable symptoms produced by it caused many to desert its use. In recent cases, he observes, the first or second application often removed the complaint; but where the cases were of long standing, it was necessary to persevere in the frictions for three or four weeks. In several cases the eruption appeared on distant parts of the body.

Dr. Robinson has lately written (in the Medical Repository, January 1821) on the good effects of the antimonial ointment rubbed on the region of the stomach in hooping-cough. By tartrite of antimony, Dr. Jenner remarks, we can not only create vesicles, but at the same time produce diseased action in the skin itself, *by deeply deranging its structure beneath the surface*—one cause, perhaps, why the sympathetic affection excited by the lytta, and those changes induced by antimony, are very different. Our author observes that, in small pox, a different degree of secondary fever will follow, when the skin is simply affected on the surface, and when it is partially destroyed beneath.

"Whoever has observed the deranged state of health where vesiculated eruptions have been called into action by an effort of Nature, must have seen how often they arrest the progress of the original disorder, and may we not from thence infer what appears to me to be a pretty general law of Nature, that she often gets rid of diseased action affecting vital organs, by exciting eruptions in other parts not vital?" 31.

Among the illustrations of this doctrine brought forward by Dr. Jenner, he mentions a remark made to him by his able and excellent friend, John Fleming, Esq. now a member of the British Senate, and formerly at the head of the medical department at Calcutta—namely, that when attacked with intermittents, and when he took the bark frequently, he seldom could get well until herpetic eruptions appeared on his lips.

That illustrious observer, Dr. Ferriar, in his medical histories and reflections, has occasionally alluded, in the most pointed terms, to the subject now before us.* "Cutaneous eruptions (he observes) often extinguish dangerous diseases." Madness and melancholy, epilepsy, delirium protracted after fever, dyspepsia, and various pulmonary affections, are all observed to be mitigated on the appearance of cutaneous disorders—especially on the return of those that, after becoming habitual, had been suddenly suppressed.

Huxham has also related some general facts that bear on this subject—and it is particularly worthy of notice that he describes the sympathy between the skin and lungs, which elucidates the favourable effect of artificial eruptions in some pulmonary disorders.

Here Dr. Jenner regrets, and with reason, that Dr. Parry's

* It is a doctrine of very old standing indeed; but in the humoral pathology, physicians considered what we now look upon as metastases of diseased *action*, to be translations of *matter*, and eruptions, abscesses, &c. were believed to be so many drains for carrying the peccant humour out of the constitution.—*Rev.*

"Elements of Pathology" is not sufficiently studied and appreciated by the profession. We hope we shall prove instrumental, in the present number of our journal, in obviating this unwarrantable apathy of our brethren to such a valuable work. At all events, we have sent forth an abstract of Dr. Parry's volume, that will be read and reflected on, where the original could never hope to travel—perhaps where it *has* travelled hitherto in vain.

In order to display, in a still stronger point of view, the analogy between the phenomena of artificial and spontaneous eruptions, Dr. Jenner draws an interesting sketch of the rise, progress, and termination of what is called an exanthematous fever—the small-pox, for example.

"Morbid animal matter, generated by this disease, is applied to the body either by what is termed the natural or artificial mode. After a given space of time, in either case, diseased action is manifested by that constitutional derangement which is designated fever. This goes on for a limited period, when eruptions appear on the skin, which soon shew on their apices vesiculated specks. Here the disease, as far as it depended on the *primary action* of the infectious matter which called it into existence, terminates. But now a new train of symptoms comes on, consequent to the diseased action excited on the skin by the *pustules*, the influence of which is felt in proportion to their numbers, their malignancy, the disposition of the constitution, and the extent to which they penetrate the skin. The fever, in the first and second instances, has two *distinct* origins. In the *first* instance, it arises from the influence of the morbid matter inhaled, or intentionally applied; in the *second*, from diseased action going forward on the skin, and, in many instances, also on the mucous membranes of the fauces, trachea, and ramifications of the bronchi. The rapidity with which, in some instances, the secondary diseased action follows the primary, often obscures the distinction. Of this the ordinary phenomena of confluent small-pox and scarlatina exhibit familiar instances. In the first of these the skin is often so quickly and universally assailed, that there is, in most instances, no interval of cessation. Nature is in a hurry to call out her guards." 40.

Here our esteemed author introduces some practical remarks on the benefits which may be derived from sedative applications, where the pustules are formed so thick upon the cutis as to augment, in a high degree, the secondary fever. An experiment was made by Mr. Fry, in the case of a young woman, who had a full burthen of distinct small-pox, and whose countenance was loaded with pustules.

"In this state one cheek was sopped with liq. lythargyri somewhat more diluted than I intended, while the other was suffered to take its course for the sake of comparison. The consequence was, that although, from excessive occupation, this process was not repeated by Mr. F. the effect was nevertheless very manifest, for the pustules were so much checked in their progress to maturation, that they could be scarcely said to have maturated at all." 40.

As Dr. Jenner rests this proposition of checking the exuberance of eruptions by sedative applications, on a single case, and that not under his own eye, we are justified in considering it as a mere speculation ; and we would hazard another speculation, namely, that the practice would prove a dangerous one. We grant, indeed, that *cool air* is always advantageous in eruptive diseases, as moderating the *eruptive* fever, and refreshing the patient; but we would be very loth to apply sedative and repellent applications to the eruptions themselves, for a similar purpose. We are sure Dr. Jenner will excuse the freedom of this remark, the propriety of which, we think, is not a little strengthened by the following observation of Dr. Jenner himself.

"Even in small-pox, though the disease itself cannot possibly disappear wholly, the eruptions, when in a vivid state of maturation, may so lose their prominent appearance, as on a sudden to become flattened and excite distress in the constitution, which is often followed by fatal consequences." 44.

Dr. Jenner remarks that, when eruptions appear early, and without the proper vesicular character, the prognosis is unfavourable. But we must pass over several pages occupied with hints, suggestions, and speculations respecting several diseases, as our limits are already exceeded.

The formula of antimonial ointment used by Dr. Jenner is the following. Ant. tart. ʒij. ungt. cetacei ʒix. sacchari albi ʒj. hydrarg. sulphur. rub. v gr. m. ft. unguentum. The tartrite of antimony should be finely levigated. The sugar preserves the ointment from rancidity. The ointment sometimes brings out crops of pustules in one day—sometimes it requires several. "In the case of a lady, where two parts of tartar emetic and one of simple cerate were used, eruptions appeared in a few hours. A communication from Mr. J. Fosbroke is inserted, in which several interesting particulars are stated relative to the action of the antimonial ointment in some cases of hysteria. A case is also introduced from the Rev. G. C. Jenner, illutrating the beneficial effects of the said ointment. We shall give it in the author's own words, as the case is short.

"William Holloway, ætat. 26,—Very tall, and of rather a spare habit, about the end of December, or beginning of January last, was attacked with violent pain in the left side, and considerable swelling about the region of the liver, with most of the usual symptoms that attend hepatitis, together with others indicative of pulmonary affection. He was bled, blistered, and took various medicines, under the direction of several medical gentlemen in the neighbourhood. These remedies afforded him a temporary relief; but he soon grew worse, and his malady continued to increase for six weeks, when I mentioned his case to you. By your advice, I furnished him with some of the ointment of tartarized antimony, and directed him to rub it on the chest. In twenty-four hours, eruptions appeared. The enlargement about the liver soon began to subside, the pain abated, and at the expiration of a month he was able to follow his usual occupation of a mason and bricklayer.

"In September last, during the unsettled weather, he went to assist a neighbour in securing his corn; when, after using great bodily exertion, and drinking freely of cider while he was very warm, he felt himself much indisposed, and in two days afterwards was seized with chills. The pain in the side returned, attended with pain on the top of the shoulder and in the chest, shortness of breath, cough, quick pulse, (I never found it under 120,) and great debility. He now used the ointment, unassisted by any internal remedy. He received it from me with the most enthusiastic rapture, and used it more profusely than I intended, not only on the chest, but on the shoulder, and wherever he felt pain. A large crop of pustules was the result, which maturated, and continued to discharge plentifully for nine days, when he was able to resume his work, and is now free from all complaint." 63.

We must now take leave of our worthy author, with the expression of our high respect for his talents, zeal, and usefulness; and with ardent wishes that the remedial process pointed out in these pages may answer the expectations of himself, and prove equally advantageous in the hands of others.

